# *FAT1* mutations cause a glomerulotubular nephropathy

**DOI:** 10.1038/ncomms10822

**Published:** 2016-02-24

**Authors:** Heon Yung Gee, Carolin E. Sadowski, Pardeep K. Aggarwal, Jonathan D. Porath, Toma A. Yakulov, Markus Schueler, Svjetlana Lovric, Shazia Ashraf, Daniela A. Braun, Jan Halbritter, Humphrey Fang, Rannar Airik, Virginia Vega-Warner, Kyeong Jee Cho, Timothy A. Chan, Luc G. T. Morris, Charles ffrench-Constant, Nicholas Allen, Helen McNeill, Rainer Büscher, Henriette Kyrieleis, Michael Wallot, Ariana Gaspert, Thomas Kistler, David V. Milford, Moin A. Saleem, Wee Teik Keng, Stephen I. Alexander, Rudolph P. Valentini, Christoph Licht, Jun C. Teh, Radovan Bogdanovic, Ania Koziell, Agnieszka Bierzynska, Neveen A. Soliman, Edgar A. Otto, Richard P. Lifton, Lawrence B. Holzman, Nicholas E. S. Sibinga, Gerd Walz, Alda Tufro, Friedhelm Hildebrandt

**Affiliations:** 1 Division of Nephrology, Boston Children's Hospital, Harvard Medical School, Boston, Massachusetts 02115, USA; 2 Department of Pharmacology, Brain Korea 21 PLUS Project for Medical Sciences, Yonsei University College of Medicine, Seoul 03722, Korea; 3 Department of Pediatrics, Yale University School of Medicine, New Haven, Connecticut 06520, USA; 4 University Freiburg Medical Center, Freiburg 79106, Germany; 5 Department of Pediatrics, University of Michigan, Ann Arbor, Michigan 48109, USA; 6 Human Oncology and Pathogenesis Program, Memorial Sloan Kettering Cancer Center, New York, New York 10065, USA; 7 MRC Centre for Regenerative Medicine, Multiple Sclerosis Society Centre for Translational Research, University of Edinburgh, Edinburgh EH16 4UU, UK; 8 School of Biosciences, Cardiff University, Museum Avenue, Cardiff CF10 3AX, UK; 9 Department of Molecular Genetics, Samuel Lunenfeld-Tanenbaum Research Institute, University of Toronto, Mount Sinai Hospital, Toronto, Ontario, Canada M5G 1X5; 10 Department of Pediatrics II, University Hospital of Essen, Essen 45147, Germany; 11 Department of Pediatrics, Bethanien Hospital, Moers 47441, Germany; 12 Institute of Surgical Pathology, University Hospital Zurich, Zurich 8091, Switzerland; 13 Division of Nephrology, Kantonsspital Winterthur, Winterthur 8401, Switzerland; 14 Department of Paediatric Nephrology, Birmingham Children's Hospital, Birmingham B4 6NH, UK; 15 Children's and Academic Renal Unit, University of Bristol, Bristol BS1 5NB, UK; 16 Department of Genetics, Hospital Kuala Lumpur, Kuala Lumpur 50586, Malaysia; 17 Centre for Kidney Research, Children's Hospital at Westmead, Westmead 2145, Australia; 18 Department of Pediatrics, Division of Pediatric Nephrology, Children's Hospital of Michigan/Wayne State University, Detroit, Michigan 48201, USA; 19 Division of Nephrology, The Hospital for Sick Children and University of Toronto, Toronto, Ontario, Canada M5G 1X8; 20 Institute for Mother and Child Health Care of Serbia “Dr Vukan Čupić”, Department of Nephrology, University of Belgrade, Faculty of Medicine, Belgrade 11000, Serbia; 21 Department of Experimental Immunobiology, Division of Transplantation Immunology & Mucosal Biology, King's College London, Faculty of Life Sciences & Medicine, 5th floor Tower Wing, Guy's Hospital, Great Maze Pond, London SE1 9RT, UK; 22 Department of Pediatrics, Center of Pediatric Nephrology & Transplantation, Kasr Al Ainy School of Medicine, Cairo University, Cairo 11562, Egypt; 23 Egyptian Group for Orphan Renal Diseases, Cairo 11562, Egypt; 24 Department of Genetics, Yale University School of Medicine, New Haven, Connecticut 06520, USA; 25 Howard Hughes Medical Institute, Chevy Chase, Maryland 20815, USA; 26 Renal-Electrolyte and Hypertension Division, Perelman School of Medicine, University of Pennsylvania, Philadelphia, Pennsylvania 19104, USA; 27 Wilf Family Cardiovascular Research Institute and Department of Medicine/Cardiology, Albert Einstein College of Medicine, Bronx, New York 10461, USA

## Abstract

Steroid-resistant nephrotic syndrome (SRNS) causes 15% of chronic kidney disease (CKD). Here we show that recessive mutations in *FAT1* cause a distinct renal disease entity in four families with a combination of SRNS, tubular ectasia, haematuria and facultative neurological involvement. Loss of *FAT1* results in decreased cell adhesion and migration in fibroblasts and podocytes and the decreased migration is partially reversed by a RAC1/CDC42 activator. Podocyte-specific deletion of *Fat1* in mice induces abnormal glomerular filtration barrier development, leading to podocyte foot process effacement. Knockdown of *Fat1* in renal tubular cells reduces migration, decreases active RAC1 and CDC42, and induces defects in lumen formation. Knockdown of *fat1* in zebrafish causes pronephric cysts, which is partially rescued by RAC1/CDC42 activators, confirming a role of the two small GTPases in the pathogenesis. These findings provide new insights into the pathogenesis of SRNS and tubulopathy, linking FAT1 and RAC1/CDC42 to podocyte and tubular cell function.

## Abstract (web summary)

Steroid-sensitive nephrotic syndrome (SRNS) can cause CKD and necessitate kidney transplant. Here the authors identify FAT1 mutations by homozygosity mapping and whole-exome sequencing in families with SRNS and provide functional mouse and zebrafish evidence that FAT1 is required for normal glomerular and tubular function and that FAT1 mutations can cause SRNS.

## Graphical abstract

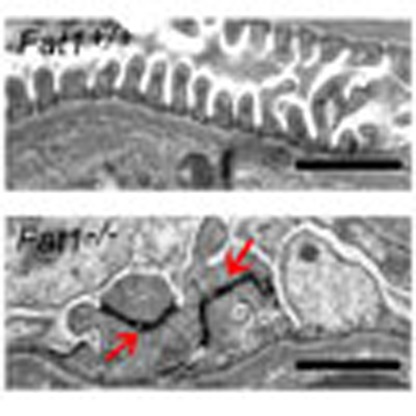

Nephrotic syndrome (NS) results in proteinuria, hypoalbuminemia and oedema, and is classified by its response to steroid treatment into steroid-sensitive NS and steroid-resistant NS (SRNS). SRNS leads to chronic kidney disease (CKD) within a few years of onset, requiring renal replacement therapy for survival. It causes 15% of all end-stage kidney disease that manifests by 25 years of age^1^. SRNS is considered one of the most intractable kidney diseases, because it has a 30% risk of recurrence after renal transplant if SRNS is idiopathic. However, in SRNS with a monogenic cause the risk of recurrence is reduced from 35 to 8% (ref. 2). Histologically, SRNS manifests as ‘focal segmental glomerulosclerosis' (FSGS) or as the early-onset developmental variant ‘diffuse mesangial sclerosis' (DMS)^3^. The first insights into the pathogenesis of SRNS were gained by the discovery of single-gene (monogenic) causes of SRNS, revealing that the encoded proteins are essential for the function of the glomerular podocyte^4^,^5^. Podocytes are neuron-like cells that extend multiple tubulin-based primary processes that branch into secondary actin-based foot processes. Foot processes interdigitate with those of neighbouring podocytes, forming between them the glomerular slit diaphragm, which is critical for the filtering process. Loss of foot process and slit membrane integrity causes glomerular protein leakage and SRNS.

Advances in molecular diagnostics enables identification of the primary cause of disease in a large fraction of individuals with SRNS. In a worldwide cohort of 1,783 families, a monogenic cause of SRNS was detected in 1 of 27 genes in ∼30% of SRNS cases manifesting before age 25 years^6^. Gene identification also enables disease modelling in cell-based and animal models of gene knockdown or knockout, facilitates screening for small molecule therapeutics and permits an aetiologic classification of SRNS for therapeutic trials.

Identification of monogenic causes of SRNS has revealed that the encoded proteins are part of protein–protein interaction complexes that functionally participate in distinct cellular tasks and signalling pathways within podocytes, including:

(i) Podocyte slit membrane signalling (NPHS1, NPHS2, CD2AP, PLCE1)^7^,^8^,^9^,^10^,

(ii) Actin-binding complexes within the foot process actin network (ACTN4, INF2, MYO1E and ANLN)^11^,^12^,^13^,^14^,

(iii) Actin regulation by RHO/RAC/CDC42 (ARHGAP24, ARHGDIA, KANK1 and 2)^15^,^16^,^17^,

(iv) Integrin/laminin signalling in podocyte focal adhesions at the basement membrane (LAMB2 and ITGA3)^18^,^19^,^20^,^21^,

(v) Coenzyme Q_10_ biosynthesis components (COQ2, COQ6, PDSS2 and ADCK4)^22^,^23^,^24^,^25^,

(vi) Transcription factors expressed in podocytes (WT1 and SMARCAL1)^21^,^26^, as well as

(vii) Calcium signalling (TRPC6)^27^,^28^ and caveolin signalling (EMP2)^29^.

Although disease mechanisms of SRNS are not well-understood, altered podocyte cell-migration rate represents a relevant assay of pathogenicity for most SRNS disease-causing alleles^16^,^30^. Actin remodelling by members of the ‘RHO family of small GTPases' RHOA, RAC1 and CDC42 (henceforth ‘RHO GTPases') regulates podocyte cell-migration rate^30^.

Because genetic mapping data indicate a multitude of additional loci-bearing mutations that might cause monogenic forms of SRNS, here we perform homozygosity mapping (HM) and whole-exome sequencing to identify novel monogenic SRNS genes. We identify mutations in *FAT1* that cause a human glomerulotubular disease with features of both SRNS and tubular ectasia. Knockdown of *Fat1* in renal tubular epithelial cells decreases cell–cell adhesion, cell migration and activity of small RHO-like GTPases RAC1 and CDC42, but also induces defects in the formation of the tubular cell lumen. Podocyte-specific *Fat1* loss-of-function recapitulates the NS phenotype in a mouse model. Furthermore, knockdown of *fat1* in zebrafish causes formation of pronephric cysts, which is partially rescued by RAC1/CDC42 activators, confirming a role of the two small GTPases in the pathogenesis of this renal disease.

Identification of this new glomerulotubular disease entity demonstrates that FAT1 is necessary for glomerular as well as tubular function. These findings provide new insights into the pathogenesis and therapeutic approaches for SRNS and tubular ectasia, and link FAT1 and RAC1/CDC42 to podocyte and tubular cell function.

## Results

### FAT1 mutations cause a glomerulotubular nephropathy

We previously showed that a causal mutation in 1 of 27 different single genes can be identified in ∼30% of cases with SRNS that manifest before 25 years of age^31^. However, genetic mapping data strongly suggest a multitude of additional monogenic causes of SRNS^32^. The finding that mutations in identified monogenic SRNS genes are very rare (*LAMB2* (refs 19, 33)*, PLCE1* (ref. 10) and *COQ6* (ref. 23) highlights the need to identify additional single-gene causes of SRNS in single affected families. To overcome this limitation, we combined whole exome sequencing (WES) with HM^32^,^34^,^35^,^36^.

**Figure 1:**
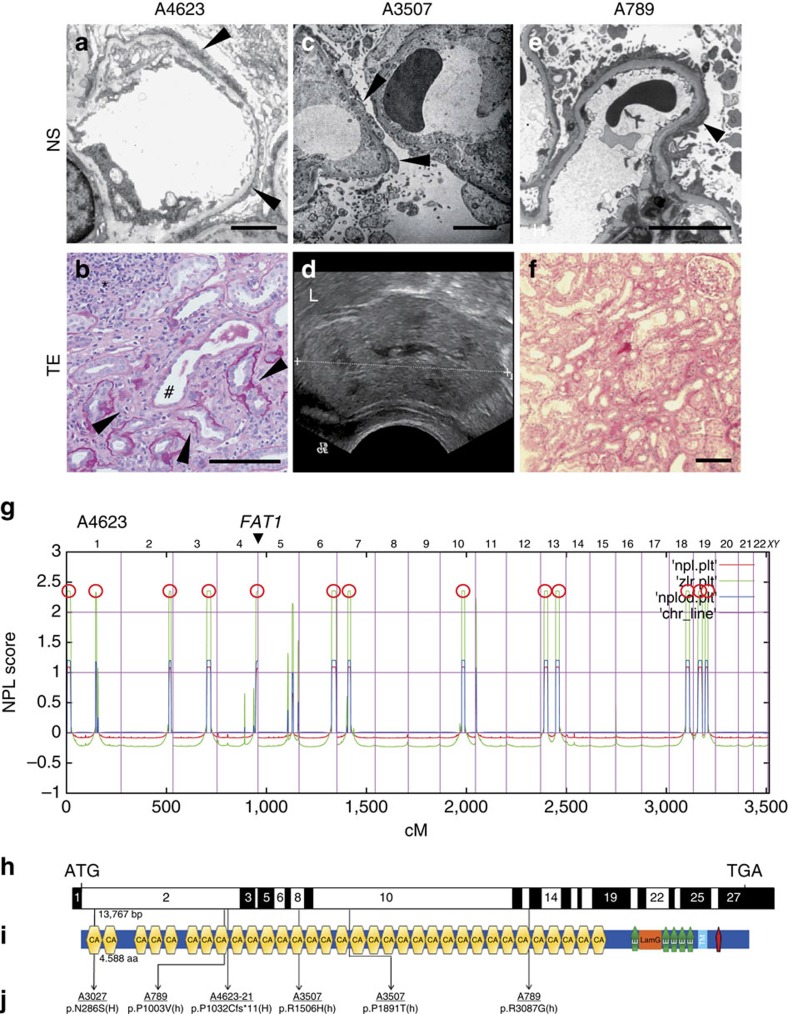
***FAT1*** **mutations cause a glomerulotubular nephropathy.** (**a**) Electron microscopy in A4623 with *FAT1* mutation demonstrates the nephrotic syndrome (NS) feature of foot process effacement (arrowheads). Scale bar, 5 μm. (**b**) Renal histology of individual A4623 exhibits cystic dilation of renal tubules (hash), interstitial infiltrations (asterisks), and tubular basement membrane disruptions (arrowheads). Scale bar, 100 μm. (**c**) In A3507 with *FAT1* mutation electron microscopy reveals the NS feature of extensive foot process effacement with microvilli formation (arrowheads). Scale bar, 5 μm. (**d**) Renal ultrasound of individual A3507 demonstrates loss of corticomedullary differentiation and increased echogenicity (L, liver). (**e**) Electron microscopy of A789 shows foot process effacement (arrowheads). Scale bar, 5 μm. (**f**) Renal histology of A789 shows tubulointerstitial infiltrates. Scale bar, 100 μm. (**g**) Homozygosity mapping identified 13 recessive candidate loci in individual A4623 with NS and tubular ectasia (TE). Non-parametric lod scores (NPL) were calculated and plotted across the human genome. The *x*-axis shows Affymetrix 250 K *Sty*I array SNP positions on human chromosomes concatenated from *p*-ter (left) to *q*-ter (right). Genetic distance is given in cM. 13 maximum NPL peaks (red circles) indicate candidate regions of homozygosity by descent. The *FAT1* locus (arrowhead) is positioned within the maximum NPL peak on chromosome 4q. (**h**) Exon structure of human *FAT1* cDNA. *FAT1* contains 27 exons. Positions of start codon (ATG) and of stop codon (TGA) are indicated. (**i**) Domain structure of FAT1. Protein domains are depicted by coloured bars in relation to encoding exon positions (**h**). *FAT1* contains 33 cadherin domains (CA), a laminin G domain (LamG) and five epidermal growth factor (EGF)-like repeat domains (green bullets) in its extracellular region, followed by a transmembrane region (light blue bar) and a C-terminal cytoplasmic domain containing a PTB-like motif (red bar) with a PDZ-binding motif (-HTEV). (**j**) Two homozygous (H) and four different compound-heterozygous *FAT1* mutations (h) detected in four families with a glomerulotubular nephropathy. Family numbers (underlined), mutations and predicted translational changes are indicated (see also Table 1).

A4623, a Turkish boy from consanguineous parents, was diagnosed with intellectual disability, pulmonary artery stenosis and bilateral blepharoptosis in early childhood. Magnetic resonance imaging showed pachygyria and Virchow–Robin spaces. He was admitted to the hospital at the age of 15 years because of proteinuria and haematuria. Kidney biopsy showed a glomerulotubular nephropathy. Typical features of SRNS, including glomerular podocyte foot process effacement (Fig. 1a), were seen together with tubular dilation, tubulointerstitial infiltrations and irregular tubular basement membranes (Fig. 1b). We performed HM in A4623. HM yielded 13 regions of homozygosity by descent that represents candidate regions for a recessive disease gene (Fig. 1g) (ref. 32). Following WES and filtering of variants from normal reference sequence, three rare variants in *FAT1*, *PIDD* and *DZIP1* remained (Supplementary Table 1) in A4623. Mutations in the 27 known SRNS genes were excluded by evaluation of the WES data. The variant in the *FAT1* gene, which encodes the FAT atypical cadherin 1, is a homozygous protein truncating mutation (p.P1032Cfs*11) (Table 1, Fig. 1h–j and Supplementary Table 1).

One child of Arab origin (A3027) presented with proteinuria and haematuria, and renal ultrasound showed increased echogenicity (Supplementary Fig. 1). A3027 was diagnosed with Ewing sarcoma, and lung and spinal metastasis at the age of 15 years. He rapidly progressed to renal failure and despite radiotherapy, chemotherapy and haemodialysis he died at the age of 19 years. Because of the rapid onset of renal failure, kidney biopsy was not performed but ultrasound showed echogenicity and urine analysis showed haematuria and proteinuria. There was no other affected individual in the family. HM of this individual yielded segments of homozygosity by descent with a cumulative genomic length of 215 Mb (Supplementary Table 1). Following variant filtering by HM and WES, two rare missense variants in *FAT1* and *EHD1* remained (Supplementary Table 1). Mutations in the 27 known SRNS genes were excluded by evaluation of the WES data. The variant (c.857A>F;p.N286S) in *FAT1* is reported as a SNP in the dbSNP database, however, its minor allele frequency is 0.0002 and it never occurred in the homozygous state (Table 1 and Fig. 1h–j). The *FAT1* variant alters an amino-acid residue conserved throughout evolution down to *Drosophila melanogaster* (Table 1).

When performing highly parallel sequencing^37^ of all *FAT1* exons in 1,500 additional individuals with features of NS and 800 individuals with features of tubulointerstitial nephroapathy, we detected in 2 additional families 4 different recessive (biallelic) mutations of *FAT1* (Table 1, Fig. 1j). In a female African-American girl (A789) from non-consanguineous parents, another compound-heterozygous mutation was detected (c.3008C>T, p.A1003V and c.9259C>G, p. R3087G). At the age of 9 years, kidney biopsy revealed minimal change NS. Urine analysis showed haematuria and proteinuria. After steroid resistance, cyclosporine A was started. One year after onset of NS, she was diagnosed with Morbus Hodgkin and chemotherapy was performed. Further information was not available and segregation analysis was not performed since the girl was lost for follow-up. There was no other affected individual in the two families. A3507, an African girl from non-consanguineous parents, showed haematuria and proteinuria at the age of 2 years. Kidney biopsy revealed DMS at the age of 2 years and she received unilateral nephrectomy at the age of 2 years. In addition, a mild hydrocephalus was drained with a ventriculoperitoneal shunt. One of the compound-heterozygous mutation (c.4517G>A, p.R1506H) was detected in her mother, but not the other. DNA from the father was not available.

Three out of four mutations are reported as a SNP in the dbSNP database, but their allele frequencies are either not available or very rare (Table 1). For exclusion of known genetic causes of SRNS, 27 known genes previously linked to SRNS were screened in these individuals, but no explanatory mutations were detected^6^. Interestingly, all affected individuals exhibited a glomerulotubular nephropathy of SRNS, tubular ectasia and microscopic haematuria (Fig. 1a–f). In addition, two individuals had a central nervous system phenotype, including hydrocephalus and pachygyria (with intellectual disability and bilateral blepharoptosis) (Table 1, Supplementary Fig. 1).

FAT1 is a member of a small family of vertebrate cadherin-like genes, designated FAT1–FAT4 in humans, whose orthologues were first recognized in *Drosophila*^38^. The FAT1 protein contains 33 cadherin repeats, followed by 5 epidermal growth factor (EGF)-like repeat domains, a laminin G domain, a transmembrane domain and an intracellular domain (Fig. 1i).

FAT cadherins play a role in cell migration, lamellipodia dynamics, cell polarity and cell–cell adhesion. Fat cadherins have been reported to interact with Ena/VASP proteins, atrophins, β-catenin, scribble and HOMER1–HOMER3, thereby influencing Wnt and Hippo signalling and the regulation of planar cell polarity (PCP), the process by which cells become polarized and organized within the plane of an epithelial sheet. The *Fat1*^*−/−*^ mouse displays abnormal podocyte foot processes, brain developmental defects and eye abnormalities^39^.

### FAT1 defects reduce cell migration and cell–cell adhesion

**Figure 2:**
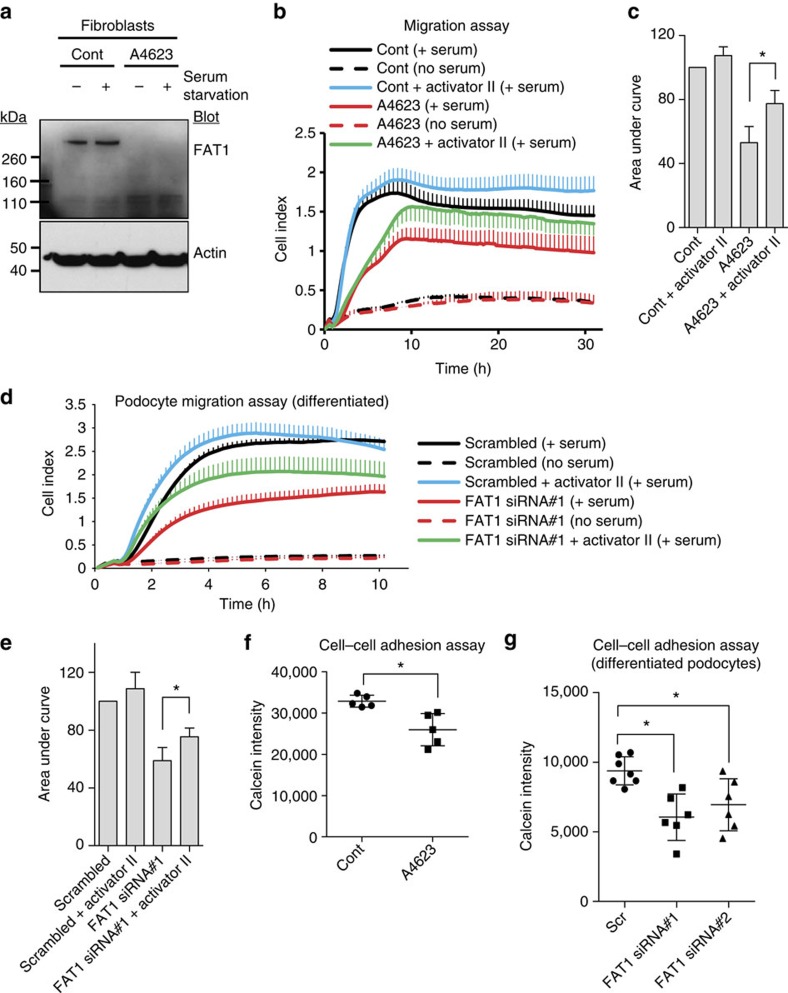
Loss of *FAT1* causes defects in migration and cell–cell adhesion in fibroblasts and cultured podocytes. (**a**) Cell lysates from fibroblasts of individuals A4623 were collected and protein level of FAT1 was analysed by western blotting with FAT1–GST antibody. FAT1–GST antibody recognizes the intracellular domain of FAT1 (C-terminal 385 aa of mouse FAT1), thus demonstrating the deficiency of the truncated FAT1 protein (p.P1032Cfs*11) resulting from c.3093_3096del *FAT1* mutation in A4623. (**b**) Cell migration assay using the xCELLigence system. Fibroblasts from A4623 show decreased migration compared with control. Note that the decrease in migration of A4623 fibroblasts was partially rescued by the RAC/CDC42 activator II. Each cell index value corresponds to the average of more than triplicates and s.d. is in only one direction for clarity. (**c**) Bar graphs represent the area under curves of **b** and data respresent the mean+s.d. of three independent experiments. *P<0.05, *t*-test. (**d**) Effect of FAT1 knockdown on podocyte migration. Differentiated cultured human podocytes transfected with FAT1 siRNA exhibited decreased migration (red line) compared with scrambled siRNA controls (black line). Decreased podocyte migration due to *FAT1* knockdown was partially rescued by RAC/CDC42 activator II (green line). Each cell index value corresponds to the average of more than triplicates and s.d. is in only one direction for clarity. (**e**) Bar graphs represent the area under curves of **d** and data respresent the mean+s.d. of three independent experiments. *P<0.05, *t*-test. (**f**) Cell–cell adhesion assay using calcein AM demonstrated that decreased cell–cell adhesion in fibroblasts from individual A4623 compared with control fibroblasts. Data respresent the mean±s.d. of more than five independent experiments in **f** and **g**. *P<0.05; *t*-test. (**g**) Knockdown of *FAT1* by two different siRNAs in differentiated podocytes resulted in decreased cell–cell adhesion in the calcein AM assay.

To examine if *FAT1* mutations cause defects of cellular function, we obtained fibroblasts from individual A4623. By immunoblotting of patient fibroblast cellular lysates (Fig. 2a) using an antibody that recognizes the intracellular domain of FAT1 (ref. 40) (Fig. 1i) we demonstrated absence of FAT1 protein in fibroblasts of individual A4623 (Fig. 2a), who carries a homozygous truncating mutation of *FAT1* (p.P1032Cfs*11) (Table 1, Fig. 1j).

To further characterize cellular defects in patient fibroblasts, we employed a cell-migration assay (Fig. 2b), which has been previously used to demonstrate deficiencies of cell migration rate in models of multiple monogenic SRNS genes, including *ARHGDIA*^16^, *KANK2* (ref. 17), *MYO1E*^13^,^41^, *ARHGAP24* (ref. 15) and *FAT1* (refs 40, 42). We found that fibroblasts from individual A4623 with a homozygous truncating mutation of *FAT1* p.P1032Cfs*11 exhibited decreased migration rate (Fig. 2b,c). The decreased migration of fibroblasts from A4623 with *FAT1* mutation was partially rescued by RAC/CDC42 activator II, indicating that RHO-like small GTPase activity may be relevant to the pathogenesis of the disease caused by loss of *FAT1* (Fig. 2b,c). Similarly, knockdown of *FAT1* in differentiated cultured podocytes led to decreased migration rate (Fig. 2d,e and Supplementary Fig. 2). This effect was likewise mediated by RHO GTPases, because decreased migration was partially mitigated by treatment with RAC/CDC42 activator II (Fig. 2d,e). To confirm whether knockdown of *FAT1* affects RHO GTPases, we used GST–rhotekin to assay the active GTP-bound state of RHOA and used GST–PAK1 to assay the active states of RAC1 and CDC42. Knockdown of *FAT1* in differentiated podocytes decreased the active state of RAC1 and CDC42 (Supplementary Fig. 3a,c,d) but had no effect on RHOA (Supplementary Fig. 3b,e), indicating RAC1 and CDC42 are more relevant to the pathogenesis of SRNS caused by loss of *FAT1*. In addition, overexpression of dominant negative CDC42 (T17N) decreased cell migration in control differentiated cultured podocytes, but failed to further decrease migration in podocytes with *FAT1* knockdown, suggesting that CDC42 is a downstream mediator of FAT1-mediated cell migration (Supplementary Fig. 4).

We then tested whether there was decreased cell–cell adhesion, which was recognized previously as a feature of *FAT1* loss-of-function^43^. We found that fibroblasts from individual A4623 with the homozygous truncating mutation exhibited decreased cell–cell adhesion (Fig. 2f). Moreover, specific knockdown of *FAT1* in differentiated cultured podocytes also impaired cell–cell adhesion, confirming that this effect is due to loss of *FAT1* expression (Fig. 2g).

### Podocyte-specific Fat1 loss-of-function leads to FSGS

**Figure 3:**
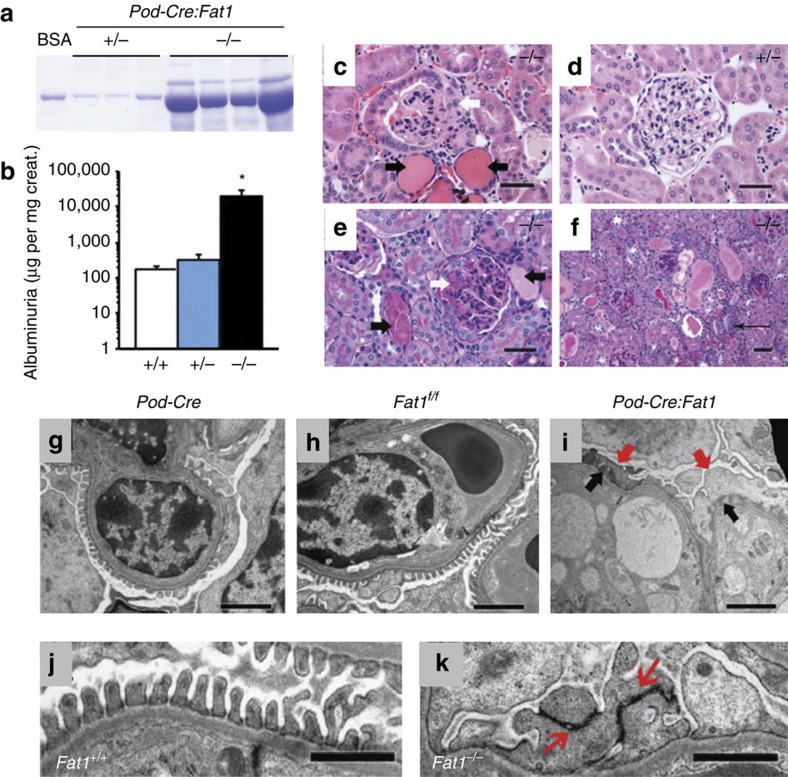
Podocyte-specific *Fat1* deletion causes massive proteinuria and FSGS in adult mice. (**a**) Urine (5 μl) resolved by SDS–PAGE and stained with Coomassie blue shows massive albuminuria in podocyte-*Fat1*^*−/−*^ mutants as quantitated in (**b**) BSA=1 μg bovine serum albumin. The number of mice studies in **a**–**c** were: *n*=10 (+/+, 5 *Pod-Cre* and 5 *Fat1*^*f/f*^); *n*=4 (*Pod-Cre:Fat1*^*+/*-^); *n*=5 (*Pod-Cre:Fat1*^*−/−*^). The experimental groups were compared by analysis of variance. *P<0.05, *t*-test. (**c**) On H&E staining, *Fat1*^*−/−*^ show focal glomerular sclerosis (white arrow) and protein casts (black arrows) compared with *Fat1*^*+/*-^ (**d**). (**e**,**f**) On PAS staining, *Fat1*^*−/−*^ kidneys show focal glomerular sclerosis (white arrows), protein casts (black arrows) and interstitial infiltrates (thin arrow). (**c**–**f**) Scale bars, 40 μm. (**g**–**h**) On TEM, *Pod-Cre* and *Fat1*^*f/f*^ single transgenic kidneys show normal glomerular filtration barrier ultrastructure. (**i**) *Fat1*^*−/−*^ kidneys exhibit complete foot process effacement (red arrows), absence of slit-diaphragms and F-actin collapse in podocytes (black arrows). GBM and endothelium are intact. (**j**) *Fat1*^*+/+*^ kidneys show normal glomerular filtration barrier ultrastructure; (**k**) In Pod-Cre:*Fat1*^*−/−*^, glomeruli tight junctions (red arrows) link the effaced foot processes. Scale bars, 2 μm (**g**–**i**); and 1 μm (**h**–**k**).

In addition to *in vitro* cell-based studies, we examined the effect of *Fat1* loss-of-function on glomerular integrity *in vivo*. Loss of *Fat1* in a constitutive *Fat1*^*−/−*^ mouse model was previously described to cause perinatal death, features of NS with podocyte foot process effacement and brain developmental defects^39^. Thus, we generated a mouse model of podocyte-specific loss of *Fat1* function, in which loss of *Fat1* is driven by the podocyte *NPHS2/podocin* promoter (Pod-Cre) (Supplementary Fig. 5a,b)^44^. Podocyte-specific *Fat1* mutants were viable, born in ratios suggesting no embryonic lethality (*n*=11 litters, 120 pups). Mice had no external malformation phenotype and survived to adulthood (up to 8 months). Newborn podocyte-specific *Fat1*^*−/−*^ mice had normal renal histology, but transmission electron microscopy (TEM) revealed persistence of cuboidal podocytes, wide foot processes and tight-junction–like cell junctions in lieu of slit-diaphragms (Supplementary Fig. 5c–k), confirming that *Fat1* is required for normal podocyte foot process and slit-diaphragm development. Adult podocyte-specific *Fat1*^*−/−*^ mutants developed progressive proteinuria with massive albuminuria at 4 months of age, ∼60-fold higher than control mice, whereas heterozygous *Fat1* mutants had only very mild albuminuria (Fig. 3a,b). However, serum albumin was not significantly different in *Fat1*^*−/−*^ and control mice (3.2±0.23 versus 3.3±0.15 g dl^−1^). Histologic examination in homozygous podocyte-specific *Fat1*^*−/−*^ mutants revealed FSGS, with the presence of protein casts and tubulointerstitial nephropathy, while only mild mesangial expansion was observed in podocyte-*Fat1* heterozygous mutants (Fig. 3c–f). On ultrastructural examination by TEM control, *Pod-Cre* and *Fat1*^*f/f*^single transgenic mice showed normal histology and ultrastructure (Fig. 3g,h,j), whereas homozygous *Fat1*^*−/−*^ mutants exhibited widespread foot process effacement, microvillar transformation and collapsed F-actin (Fig. 3i,k). Slit-diaphragms are remarkably decreased in *Fat1*^*−/−*^ mutants and cell junctions of effaced foot processes resemble tight junctions (Fig. 3k).

### Loss of Fat1 causes CDC42-mediated renal tubular defects

**Figure 4:**
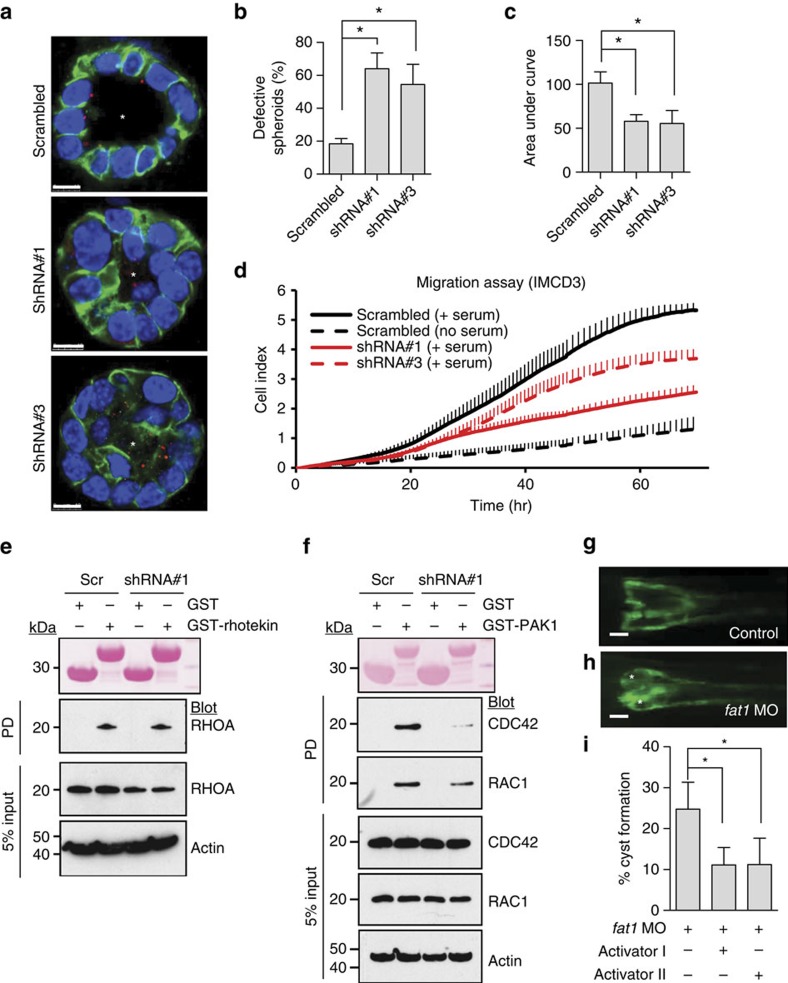
Loss of *FAT1* causes a renal tubular phenotype via defective RHO GTPase signalling. (**a**) IMCD3 cells ciliated apically and formed a spheroid containing a central lumen (asterisk) when grown in 3D matrigel culture for 3 days; lumens were perturbed on *Fat1* knockdown (shRNA#1 and shRNA#3). Cells were stained for acetylated α-tubulin (red), β-catenin (green) and DAPI (blue). Scale bars, 10 μm. (**b**) Percentage of abnormal spheroids. More than 50 spheroids were examined in each experiment. Data represent the mean+s.d. of three independent experiments in **b**,**c**. *P<0.05, *t*-test. (**c**) The effect of Fat1 knockdown on cell migration. Bar graphs represent the area under curves of **d**. (**d**) The effect of *Fat1* knockdown on cell migration. Compared with baseline (dashed black line), addition of serum strongly increases migration rate in IMCD3 cells with scrambled shRNA (Scr) (solid black line). In contrast, IMCD3 cells stably transfected with *Fat1* shRNAs #1 (red continuous line) or #3 (red dashed line) exhibited slower rate of migration. Each cell index value corresponds to the average of more than triplicates and s.d. is in only one direction for clarity. (**e**) Active GTP-bound RHOA precipitated from IMCD3. Cells transfected with scrambled control siRNA versus *Fat1* shRNA exhibited no significant difference in relative RHOA activity. This is representative of three experiments. (**f**) Active GTP-bound CDC42 or RAC1 using a GST–PAK1 (CRIB) pull-down assay. Note that *Fat1* knockdown leads to a significant decrease in relative CDC42 and RAC1 (31% and 44%, respectively) compared with Scr control cells. This is representative of four experiments. Quantification of **e** and **f** is presented in Supplementary Fig. 7. (**g**–**h**) *fat1* morpholino-oligonucelotide (MO) was injected to Wt1b::GFP transgenic zebrafish. Zebrafish injected with control MO did not produce any phenotype. Depletion of *fat1* by a *fat1* MO targeting the translation initiation site of zebrafish *fat1* caused pronephric cysts (asterisks) in 78 of 325 zebrafish embryos (24%). Scale bars, 100 μm. (**i**) Activator I (Rho/Rac/Cdc42 activator I) reduced cyst formation to 9.7% (26 of 268 embryos), and activator II (Rac/Cdc42 activator II) to 12.0% (59 of 490 embryos). *P<0.001, *χ*^2^-test.

To examine mechanisms of tubular defects of renal tubule architecture, we performed transient *Fat1* knockdown in mouse inner medullary collecting duct (IMCD3) cells, a renal tubule cell line that has broadly been used to study tubular defects^35^,^45^. We found that knockdown of *Fat1* perturbed lumen formation of IMCD3 spheroids (Fig. 4a,b), suggesting that FAT1 is involved in the regulation of apicobasal polarity. However, this lumen formation did not result from ciliogenesis because cells with *FAT1* knockdown did not have a problem in ciliation (Fig. 4a). Similarly, fibroblasts from A4623 with a homozygous truncating mutation did not show any defect in number or structure of cilia compared with control fibroblasts (Supplementary Fig. 6), suggesting that FAT1 is not directly involved in ciliogenesis.

Because fibroblasts of individuals with *FAT1* mutation had cell migration defects (Fig. 2b), and because Fat1 also regulates cell migration in many different cell types including podocytes, we examined whether Fat1 also regulates cell migration in IMCD3 renal tubular cells. We found that knockdown of *Fat1* using two different short hairpin RNAs (shRNAs) did in fact decrease cell migration rate in IMCD3 cells (Fig. 4b,c and Supplementary Fig. 7a).

Apicobasal cell polarity is controlled by the PCP pathway and FAT family proteins are involved in this pathway^46^. FAT family proteins are upstream PCP proteins, Scribble is one of the PCP core proteins and RHO GTPases are downstream PCP effector proteins^47^. Therefore, we hypothesized that the observed defects in both lumen formation and cell migration may be mediated by disruption of RHO/RAC/CDC42 signalling. When measuring active RHOA by a GST–Rhotekin pull-down assay in IMCD3 cells, we found that on knockdown of *Fat1*active GTP-bound RHOA was unaltered (Fig. 4e and Supplementary Fig. 7b). In contrast, when measuring active CDC42 and RAC1 by GST–PAK1 pull-down assay, we found that on knockdown of *Fat1* active CDC42 and RAC1 were both decreased (Fig. 4f and Supplementary Fig. 7c,d). These results in IMCD3 cells are congruent with those in cultured podocytes (Supplementary Fig. 3) and indicate that CDC42 and RAC1 are more relevant than RHOA for the pathogenesis of the glomerulotubular disease that is caused by loss of *FAT1*.

We then studied *in vivo* the effects on renal tubule cell architecture that we had observed in cell-based systems *in vitro*. We chose zebrafish as a model organism, because zebrafish embryo is amenable to genetic manipulation such as injection of morpholino (MO) and knockdown of *fat1* in zebrafish is known to cause pronephric cysts^47^. We performed MO knockdown of *fat1* in Wt1b::GFP transgenic zebrafish, in which pronephric kidney cysts can readily be visualized. We found that, whereas zebrafish injected with control MO did not produce any phenotype (Fig. 4e), depletion of *fat1* caused pronephric cysts in 24% of zebrafish embryos (Fig. 4f). To further corroborate the pathogenic role of RAC1/CDC42 signalling, we tested the effect of two different RHO/RAC/CDC42 activators on rescue of the zebrafish pronephric renal cystic phenotype. We found that the chemically distinct Rho GTPase activators I and II reduced cyst formation from 31% to 9.7% and 12.0%, respectively (Fig. 4i).

## Discussion

We here identify recessive mutations of *FAT1* as causing a glomerulotubular nephropathy. Four families with FAT1 mutations presented with a combination of SRNS, tubular ectasia, haematuria and facultative neurological involvement. Mechanistically, our studies implicate RHO-like small GTPase signalling in the pathogenesis of both, the glomerular and the tubular phenotypes.

In a global *Fat1* knockout mice, *Fat1* null pups died within 48 h of birth, which was attributed to abnormal podocyte foot process fusion with obliteration of the slit membranes^39^. These findings are consistent with the concept that FAT1 provides spacing between cells in the kidney and plays a role in the formation of intercellular junctions in the kidney^60^. While our model of podocyte-specific *Fat1* deletion caused identical podocyte foot process fusion at birth, we did not observe perinatal lethality, suggesting that perinatal lethality with global *Fat1* loss reflects a different developmental problem.

*Fat1* null mice also exhibited partially penetrant midline developmental defects including holoprosencephaly, as expected from strong *Fat1* expression within the neuroepithelium.^39^ This mouse model is consistent with our finding of a combined renal and neurologic disease phenotype in human recessive *FAT1* mutation. Our observation of incomplete penetrance of the neuronal involvement in individuals with *FAT1* mutation parallels the findings in the mouse model and is most likely due to compensation by the other FAT proteins (FAT2, 3 or 4)^38^, and to the fact that all of the individuals with two recessive *FAT1* mutations (except A4623) carried at least one hypomorphic allele, that is, an allele that is expected not to convey full loss-of-function (Table 1). It is also consistent with the finding that individual A4623, who carries the homozygous truncating mutation, has a disease phenotype with the most severe neuronal involvement (Table 1).

Cooperation of different members of FAT cadherins was demonstrated in mouse models of combined loss-of-function of *Fat4* and *Fat1*, where FAT proteins cooperate in regulating multiple aspects of tissue morphogenesis in the kidney and other organs. Fat function affected renal tubular elongation, neural tube formation and cochlear morphology via its effects on mechanisms of PCP. In this context, removal of one copy of the murine *Fat1* gene exacerbated the renal tubular cyst phenotype of mice^48^.

Taken together, our cell-based and *in vivo* studies using mouse and zebrafish suggest that the glomerular SRNS-like disease phenotype results from impaired cell migration of glomerular podocytes. Indeed, defective podocyte migration is a disease mechanism common to multiple different monogenic forms of SRNS^30^. Likewise, defective cell migration was recently recognized as a central mechanism of renal tubular regeneration^49^. Interestingly, *Fat1* knockdown limited migration of renal tubule cells and also impaired their ability to form lumens in cultured spheroids, suggesting that also the renal tubular defect may be mediated by impaired cell migration.

We further demonstrated that RAC1 and CDC42 activity are impaired in both cultured podocytes and tubular cells with decreased *Fat1* expression, and that activation of these small GTPases partially corrects defective cellular migration that occur with *Fat1* deficiency. This suggests that the pathogenesis of both the renal glomerular and tubular defects is mediated by decreased RAC1/CDC42 as a potential unifying mechanism underlying both the glomerular and tubular disease phenotypes. We confirm the relevance of this disease mechanism by showing that two different RAC1/CDC42 activators mitigate the pronephric cyst phenotype in zebrafish.

Data from human genetics and animal models have shown the importance of RHO GTPase signalling in CKDs^15^,^16^,^50^,^51^,^52^. *ARHGAP24* or *ARHGDIA* mutations identified in human SRNS cause upregulation of both RAC1 and CDC42 and lead to increased podocyte migration^15^,^16^. This is congruent with the finding that active RAC1 (but not RHOA) is upregulated in *Arhgdia*^*−/−*^ mice. However, CDC42 activity has not been examined in the *Arhgdia*^*−/−*^ mouse model^53^. Scott *et al*.^50^ showed that podocyte-specific deletion of CDC42 (but not RHOA and RAC1) in mice results in foot process effacement and proteinuria. *INF2* mutations also decrease active CDC42 at the plasma membrane and cause mislocalization of CDC42 within the cytoplasm^52^. Therefore, either aberrant activation or inactivation of CDC42 interferes with podocyte function. In addition, *cdc42* knockdown in zebrafish led to hydrocephalus, body oedema and pronephric cysts, and kidney tubule-specific deletion of *CDC42* resulted in renal cysts and renal failure within weeks of birth^51^, indicating that CDC42 is also important for proper tubular function. In this study, we show that the impaired podocyte migration and the pronephric cysts in *fat1*-knockdown zebrafish were partially rescued by a RAC/CDC42 activator, suggesting that the combined glomerulotubular phenotype resulting from *FAT1* mutations is mediated by decreased RAC1 and CDC42 in both glomeruli and tubules.

Recently Morris *et al*.^43^ identified *FAT1* mutations in human cancers including glioblastoma and colorectal cancer, and showed that loss of *FAT1* leads to aberrant activation of Wnt signalling. The *FAT1* mutations identified by Morris *et al*.^43^ were somatic and mostly heterozygous. However, in the glomerulotubular disease described here renal disease resulting from *FAT1* mutations is recessive, requiring two mutant alleles, which are inherited from the heterozygous parents. It is not clear whether individuals with recessive *FAT1* mutations will develop cancer later in life. However, it is important to note that two individuals (A3027 and A789) had Ewing sarcoma and Hodgkin's lymphoma, respectively (Table 1). How the respective *FAT1* mutations identified in these individuals might contribute to neoplastic disease will require further focused study.

FAT1 is implicated in Hippo signalling through the interaction with Scribble and Hippo signalling is required for normal pronephros development in zebrafish^47^. Yes-associated protein (Yap) is a downstream transcriptional effector of Hippo signalling and is essential for nephron induction and morphogenesis^54^. Recently, it was shown that Cdc42 acts upstream of Yap during development to promote Yap-dependent gene expression and shape functioning nephrons^54^. Therefore, it is also conceivable that the glomerulotubular nephropathy caused by loss of FAT1 may result from defective Hippo signalling due to decreased Cdc42 activity, which we have shown in this study (Fig. 4f and Supplementary Fig. 3a).

Because SRNS cause ∼15% of all CKD in the first two decades of life^6^, the balance of RAC1/CDC42 signalling may be a worthwhile target when developing drugs to prevent ESKD that is caused by SRNS.

## Methods

### Study participants

Following informed consent for WES, we obtained clinical data and blood samples from individuals with SRNS or NPHP from worldwide sources. Approval for human subjects' research was obtained from the University of Michigan and Boston Children's Hospital Institutional Review Boards and from relevant local Review Boards. Deposition of WES data in public databases was not obtained at the time. The diagnosis of NS or NPHP was made by (paediatric) nephrologists based on standardized clinical and renal histologic criteria^55^. Renal biopsies were evaluated by renal pathologists. Clinical data were obtained using a standardized questionnaire (http://www.renalgenes.org).

### Whole exome sequencing

We combined WES with HM as established previously^32^,^36^. For HM^32^ the ‘Human Mapping 250 k *Sty*I' array (Affymetrix) was utilized. Genomic DNA samples were hybridized, and scanned using the manufacturer's standard protocol at the University of Michigan Core Facility (www.michiganmicroarray.com). Non-parametric logarithm of odds (LOD) scores were calculated using a modified version of the program GENEHUNTER 2.1 (refs 56, 57) through stepwise use of a sliding window with sets of 110 SNPs using the program ALLEGRO^58^. Genetic regions of homozygosity by descent (‘homozygosity peaks') were plotted across the genome as candidate regions for recessive disease-causing genes as described^32^,^35^. Disease allele frequency was set at 0.0001, and Caucasian marker allele frequencies were used. Variant burden analysis was performed as previously described^59^ using Agilent SureSelect human exome capture arrays (Life technologies) with next-generation sequencing on an Illumina sequencing platform. Sequence reads were mapped against the human reference genome (NCBI build 37/hg19) using CLC Genomics Workbench (version 6.5.1) software (CLC bio, Aarhus, Denmark). Mutation calling (Supplementary Table 1) was performed by geneticists/cell biologists, who have knowledge of clinical phenotypes, pedigree structure, HM and WES evaluation. All the coding exons of *FAT1* were examined by applying a high-throughput mutation analysis method of microfluidic array-based muyltiplex PCR (Fluidigm 48.48 Access Array) and consecutive barcoded next-generation sequencing (MiSeq, Illumina)^37^.

### Zebrafish maintenance and injections

Approval for zebrafish research was obtained from the University Committee on the Use and Care of Animals (UCUCA) of the University of Freiburg. Transgenic *wt1b::GFP* zebrafish line was raised and maintained as described^60^. Fertilized eggs were microinjected with 4 nl of injection solution at the one-to-two-cell stage with MOs (Gene Tools LLC) diluted in 200 mM KCl, 0.1% Phenol Red (Sigma-Aldrich Corporation) and 10 mM HEPES. *zFat1* MO has been previously described^47^. About 0.5 pmol of zebrafish p53 MO (5′- GCGCCATTGCTTTGCAAGAATTG -3′, Gene Tools) was always co-injected to reduce the unspecific effects of the MOs^61^. About 1.6 pmol *zFat1* MO was injected either alone or together with either 1 μg ml^−1^ of Rho/Rac/Cdc42 Activator I or 0.5 U ml^−1^ of Rac/Cdc42 activator II (Cytoskeleton). Scoring of percentage of cyst formation and zebrafish embryo imaging was done on mixed male and female embryos at 48 h post fertilization under a Leica MZ16 stereo microscope (Leica).

### Podocyte conditional *Fat1*-deficient mice

To develop the *Fat1* conditional allele in mice, homologous recombination was used to introduce *LoxP* sites flanking exon 2. The targeting construct for the *Fat1* locus was generated from a C57/BL6 strain bacterial artificial chromosome (BAC) clone (Children's Hospital Oakland Research Institute) by homologous recombination BAC engineering techniques (Open Biosystems). All junctions and overall structure were confirmed by sequencing and long-range PCR. *LoxP* sites were introduced on each side of exon 2, which encodes the signal peptide and N-terminal 1,089 aa of Fat1. The neomycin selection cassette was flanked by *Frt* sequences for removal by Flp recombinase.

Electroporation of the linearized construct into WW6 embryonic stem (ES) cells, followed by selection with G418 and ganciclovir, yielded a high percentage (∼60%) of homologous recombinants. A *Fat1*^*+/lox*^ES clone injected into C57/BL6 blastocysts yielded three highly chimeric mice that transmitted the targeted allele to offspring. The *Frt*-flanked neo cassette was removed in a cross with a FLPeR mouse (129S4/SvJaeSor*Gt (ROSA) 26Sor*^*tm1(FLP1)Dym*^/J, Jackson Labs). Heterozygous mice were interbred to generate homozygous conditionally targeted Fat1 mice (*Fat1*^*loxP/loxP*^) in a mixed 129SV/BL6 genetic background. To delete *fat1* selectively in podocytes, female *Fat1*^*+/fl*^ mice were bred with male *podocin-cre* mice^44^ and double heterozygous were backcrossed to obtain *pod-cre:Fat1*^*fl/fl*^. *Podocin-cre* mice were provided by Dr Holzman. All animals were housed in pathogen-free conditions, and protocols approved by the standing committees of the Albert Einstein Institute for Animal Studies and the Yale Animal Resources Center. Urinary albumin was measured using mouse albumin ELISA (Bethyl Laboratories) and SDS–PAGE/Coomassie Blue staining as previously described^62^. Plasma and 24 h urine creatinine were measured by HPLC^62^. Renal morphology was examined in newborn (1–3-day-old) mice and in 4–8-month-old mice by light microscopy (haematoxylin and eosin- and periodic acid-Schiff-stained sections) and by TEM using standard techniques^62^.

### Cell culture and transfection

The immortalized human podocytes^63^ were kindly provided by Dr Saleem (the University of Bristol) and maintained at the permissive temperature of 33 °C in RPMI+GlutaMAX-I (Gibco) supplemented with 10% fetal bovine serum, penicillin (50 IU ml^−1^)/streptomycin (50 μg ml^−1^) and insulin–transferrin–selenium-X. To differentiate podocytes, they were cultured at 37 °C for 14 days. The FAT1-specific and control scrambled short interfering RNAs (*siRNAs)* were purchased from Dharmacon. siRNAs were transfected into podocytes using Lipofectamine RNAiMAX (Invitrogen). Mouse kidney collecting duct IMCD3 cells were obtained from the ATCC (CRL-2123) and cultured in DMEM/F12 with 10% fetal bovine serum and penicillin (50 IU ml^−1^)/streptomycin (50 μg ml^−1^). To achieve stable knockdown of FAT1, IMCD3 cells were transducted with lentivirus, which contained shRNAs. The target sequences of siRNAs and shRNAs used in this study are in Supplementary Table 2. The shRNA-stable IMCD3 cells were selected and maintained with 8 μg ml^−1^ puromycin. Human fibroblasts were grown in DMEM supplemented with 15% FBS, penicillin (50 IU ml^−1^)/streptomycin (50 μg ml^−1^) and non-essential amino acids (Invitrogen). Rho/Rac/Cdc42 activator I and Rac/Cdc42 activator II were purchased from the Cytoskeleton, Inc.

### Lentiviral overexpression

The coding sequence of CDC42-T17N was subcloned to pLenti-CMV-Blast vector (Addgene plasmid #17451)^64^. For overexpression in differentiated cultured podocytes, HEK293T cells were transfected with pLenti-CMV-Blast-CDC42-T17N and lentiviral packaging vectors, pRSV-Rev, pMDLg/pRRE and pMD2.G. The lentivirus-contacting medium was collected after 48 h and podocytes were infected on day 12 of differentiation with 8 μg ml^−1^ polybrene-containing virus media. Podocytes were used for migration assay for 72 h after infection.

### Immunoblotting and GST pull-down assay

These experiments were performed as described previously^65^. Anti-acetylated-α-tubulin (acetyal K40), Anti-β-actin (AC-15) (Abcam), anti-Rac1 (102/Rac1), anti-Cdc42 (44/CDC42), anti-β-catenin (14/Beta-Catenin) (BD Transduction Laboratories), anti-RhoA (26C4) (Santa Cruz Biotechnology) and CEP164 (13) (Sigma-Aldrich) were purchased from commercial sources. The FAT1–GST antibody, which was generated against C-terminal 385 aa of mouse FAT1, was previously described by Moeller *et al*^40^. All of the antibodies were used at 1:1,000 for immunoblotting. Uncropped scans of the western blots are shown in Supplementary Fig. 8.

### Migration assay

Real-time migration assay was performed using the xCELLigence system (ACEA Biosciences) in CIM-plate 16 according to the manufacturer's instruction. Briefly, 4 × 10^4^ cells of fibroblasts, cultured human podocytes or IMCD3 were plated in serum-free media in the upper chamber. The lower chambers were with 10% FBS for chemoattraction or with serum-free media. The obtained data were analysed using the RTCA software. Results are presented as the time versus cell index curve.

### Spheroid assay

IMCD3 cells were trypsinized and resuspended cells were then mixed 1:1 with growth factor-depleted matrigel (BD Bioscience). This mixture was then moved to the well of a Nunc Lab-Tek II chambered coverglass (Thermo Fisher) and allowed to polymerize (45 min to 1 h) at 37 °C. Once the matrigel was fully solidified, warm medium was added drop-wise to the matrix until covered. After 72 h, the cells had formed spheroids with visible cleared lumens. The matrigels were then fixed in fresh 4% PFA for 30 min at room temperature and subsequently permeabilized for 15 min in gelatin-dissolving PBS and 0.5% Triton X-100. The primary antibodies (Anti-acetylated-α-tubulin 1:2,000 and β-catenin, 1:500) were incubated at 4 °C overnight. Fluorescent images were obtained with a Leica SP5X laser scanning microscope.

### Fluorescence-based cell–cell adhesion assay

This assay was performed as described previously^43^. Fibroblasts or cultured human podocytes were resuspended at 1 × 10^6^ cells per ml in serum-free medium supplemented with 5 μM calcein AM and incubated at 37 °C for 30 min. Cells were washed twice with serum-free medium, and 1 × 10^5^ cells were added to microplate wells containing confluent (unlabelled) cells. Calcein-labelled cells were allowed to adhere for 45 min at 37 °C. Non-adherent calcein-labelled cells were washed away with medium, and PBS was added to each well. Fluorescence was measured at an absorbance of 494 nm and emission of 517 nm using a SpectraMax Multilabel Microplate Reader (Molecular Devices).

## Additional information

**How to cite this article:** Gee, H. Y. *et al*. *FAT1* mutations cause a glomerulotubular nephropathy. *Nat. Commun.* 7:10822 doi: 10.1038/ncomms10822 (2016).

## Supplementary Material

Supplementary Information Supplementary Figures 1-8 and Supplementary Table 1 – 2.

## Figures and Tables

**Table 1:** Mutations of *FAT1* in four families with nephrotic syndrome (NS) and tubular ectasia (TE)

**Family-Individual**	**Sex**	**Ethnic origin**	**Nucleotide change**	**Amino-acid change**	**Exon (zygosity, segregation)**	**MT**	PP2*	**Amino-acid conservation**	**Frequencies in the EVS database**†	**Frequencies in the dbSNP database**‡	**Consanguinity**	**Age of onset (age at ESRD)**	**Renal manifestation**	**Extrarenal manifestations**	**Biopsy (at age)**	**Therapy and response**
A4623	M	Turkish	c.3093_3096del	p.P1032Cfs*11	2 (Hom)	NA	NA	*—*	*—*	—	Yes	15 years	NS, TE, HU	ID, PMG, BP, PAS	TIN, MS, thin GBM (12 years)	—
A3027	M	Arab	c.857A>G	p.N286S	2 (Hom)	DC	0.016	*D.r.*	*—*	rs201488687 MAF=0.0002	Yes	15 years (15 years)	NS, TE, HU	Ewing sarcoma (15 years) with lung and spinal metastasis, RVUR III^o^	ND	HD, RT, CHT, deceased at 19 years
A789	F	African-American	c.3008C>Tc.9259C>T	p.A1003Vp.R3087G	2 (het)13 (het)	DCDC	10.999	*C.e.C.e.*	AA=0/AG=1/GG=6110AA=0/AG=1/GG=6,164	rs369363545 (MAF, N/A)rs375998390 (MAF, N/A)	No	9 years	NS, TE, HU	Hodgkin lymphoma (10 years)	MCNS (9 years)	SR, CsA, CHT
A3507	F	African	c.4517G>Ac.5671C>A	p.R1506Hp.P1891T	8 (het)10 (het)	DCDC	0.4941.0	*C.e.C.e.*	*—*TT=0/TG=2/GG=6,048	*—*rs185078412MAF=0.0006	No	2 months	NS, TE, HU	Hydrocephalus	DMS (2 years)	Unilateral NE (2 years)

BP, Bilateral blepharoptosis; *C.e.*, *Caenorhabditis elegans*; CHT, chemotherapy; CsA, cyclosporin A; DC, disease causing; DMS, diffuse mesangial sclerosis; *D.r., Danio rerio*; ESRD, end-stage renal disease; EVS, Exome Variant Server; F, female; FSGS, focal segmental glomerulosclerosis; GBM, glomerular basement membrane; HD, haemodialysis; het, heterozygous; Hom, homozygous; HU, haematuria, ID, Intellectual disability; m, Maternal; M, male; MCNS, minimal change nephrotic syndrome; mo, month; MS, mesangial sclerosis; MT, mutationtaster; N, no; NA, not applicable; N/A, not available; NE, nephrectomy; ND, no data; p, paternal; PAS, pulmonary artery stenosis; PMG, pachygyria; PP2, Polyphen Prediction score Humvar; RT, radiotherapy; RVUR, right vesicourethral reflux; SR, steroid resistant; TE, tubular ectasia; TIN, tubular interstitial nephritis; Y, yes.

^*^Polyphen prediction score HumVar ranges from 0 to 1.0. 0, benign; 1.0, probably damaging.

^†^Exome variant Server (http://evs.gs.washington.edu/EVS/).

^‡^dbSNP database (http://www.ncbi.nlm.nih.gov/SNP).
