# Supplementary material for: FAT1 mutations cause a glomerulotubular nephropathy
Source: Nat Commun. 2016 Feb 24;7:10822. doi: 10.1038/ncomms10822 (PMC4770090; doi:10.1038/ncomms10822)
Supplement: Supplementary Information — Supplementary Figures 1-8 and Supplementary Table 1 – 2. [file ncomms10822-s1.pdf]

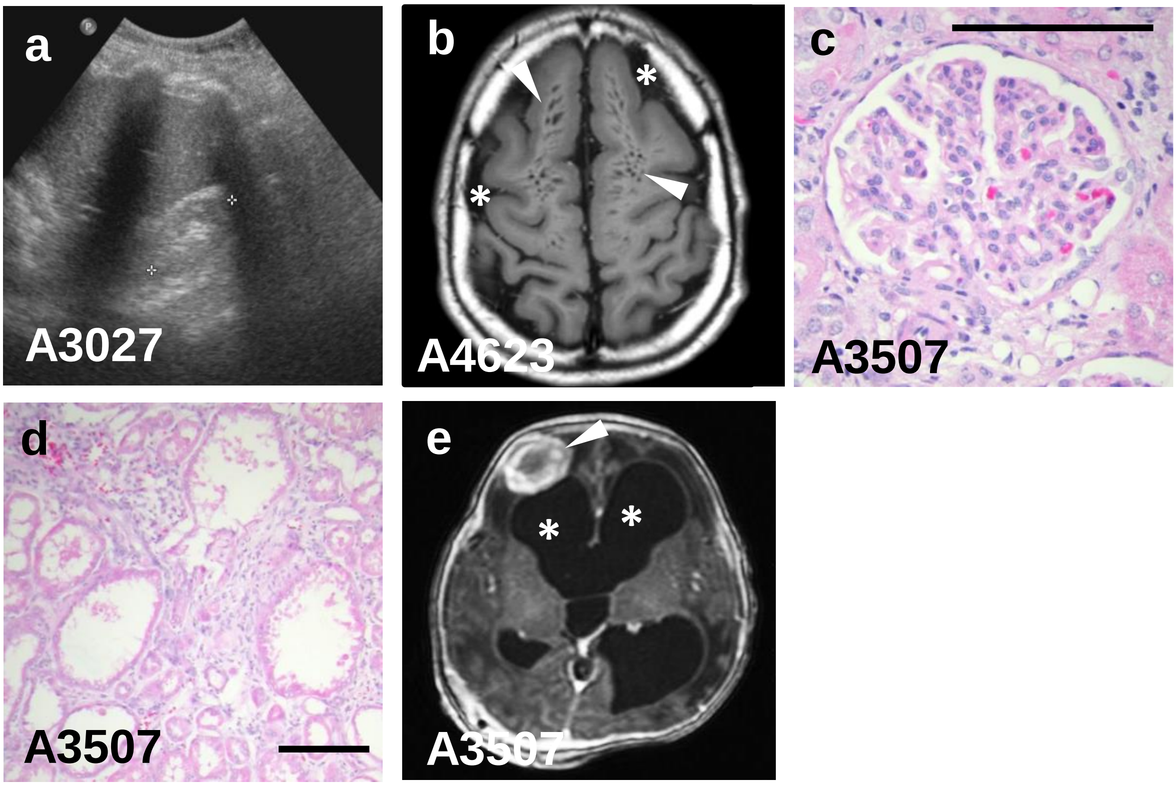

Supplementary Figure 1. Clinical manifestations of individuals with *FAT1* mutations.

(a) Renal ultrasound of right kidney in A3027 reveals small renal cysts, loss of corticomedullary differentiation, and increased echogenicity, rendering the renal pattern brighter than the liver's (L).

(b) Cranial MRI of A4623 depicts pachygyria with few broad coarse gyri (asterisks) and dilated Virchow-Robin spaces (arrowheads).

(c) Renal histology of A3507 shows diffuse mesangial sclerosis (H&E stain). Scale bar, 100 μm.

(d) Renal histology of A3507 shows tubular cysts, interstitial fibrosis and infiltrations. Scale bar, 100 μm.

(e) Brain MRI of A3507 after VA shunt implantation in the right ventricle shows dilation of the lateral ventricles (asterisks) and an area of hemorrhage (arrowhead).

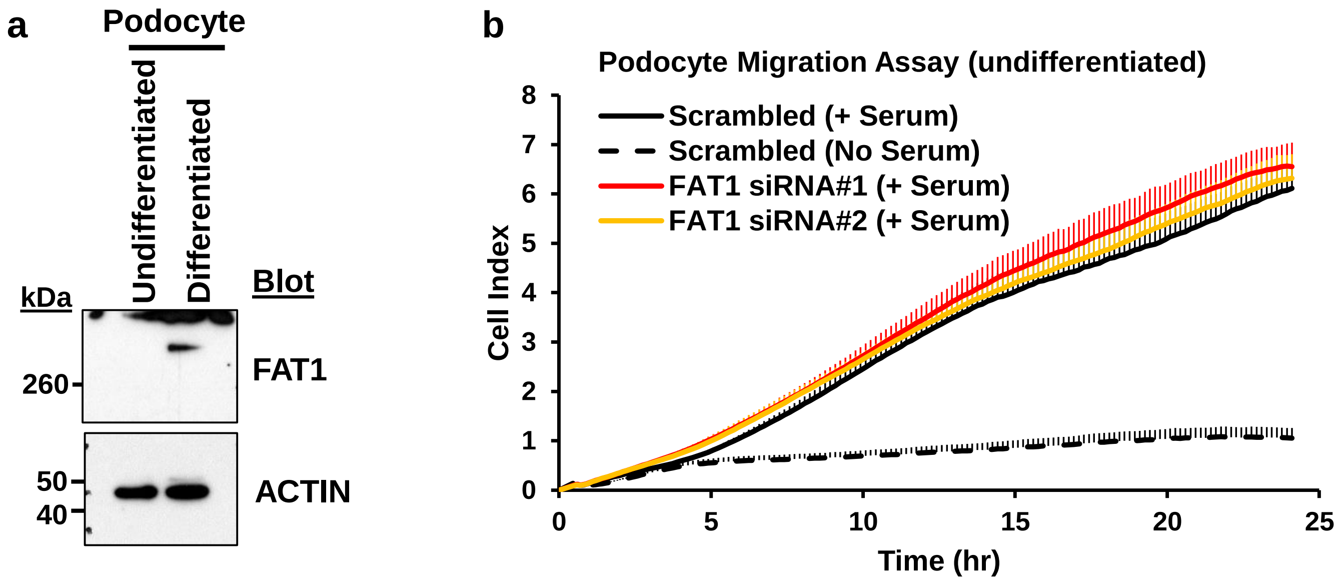

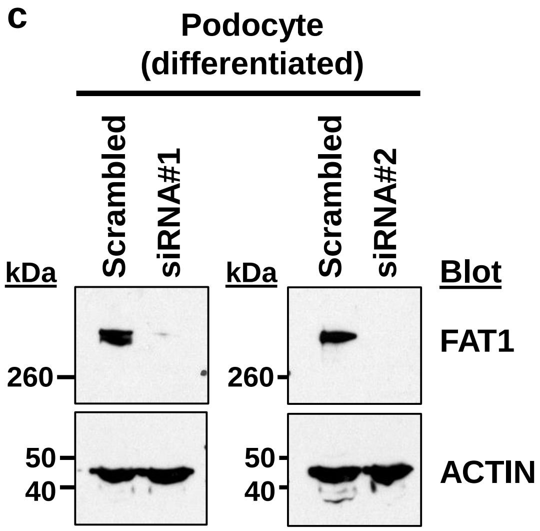

Supplementary Figure 2. *FAT1* knockdown in cultured human podocytes and in IMCD3 renal epithelial cells.

(a) Undifferentiated cultured human podocytes do not express FAT1 endogenously, whereas differentiated cultured podocytes express FAT1.

(b) FAT1 siRNAs did not have any effect on migration in undifferentiated podocytes because undifferentiated podocytes do not express FAT1. Error bars are shown in one direction only for clarity and indicate SDs for e indipendent expreiments.

(c) FAT1 knockdown by siRNAs (siRNA#1 and siRNA#2) in differentiated podocytes significantly reduced FAT1 protein.

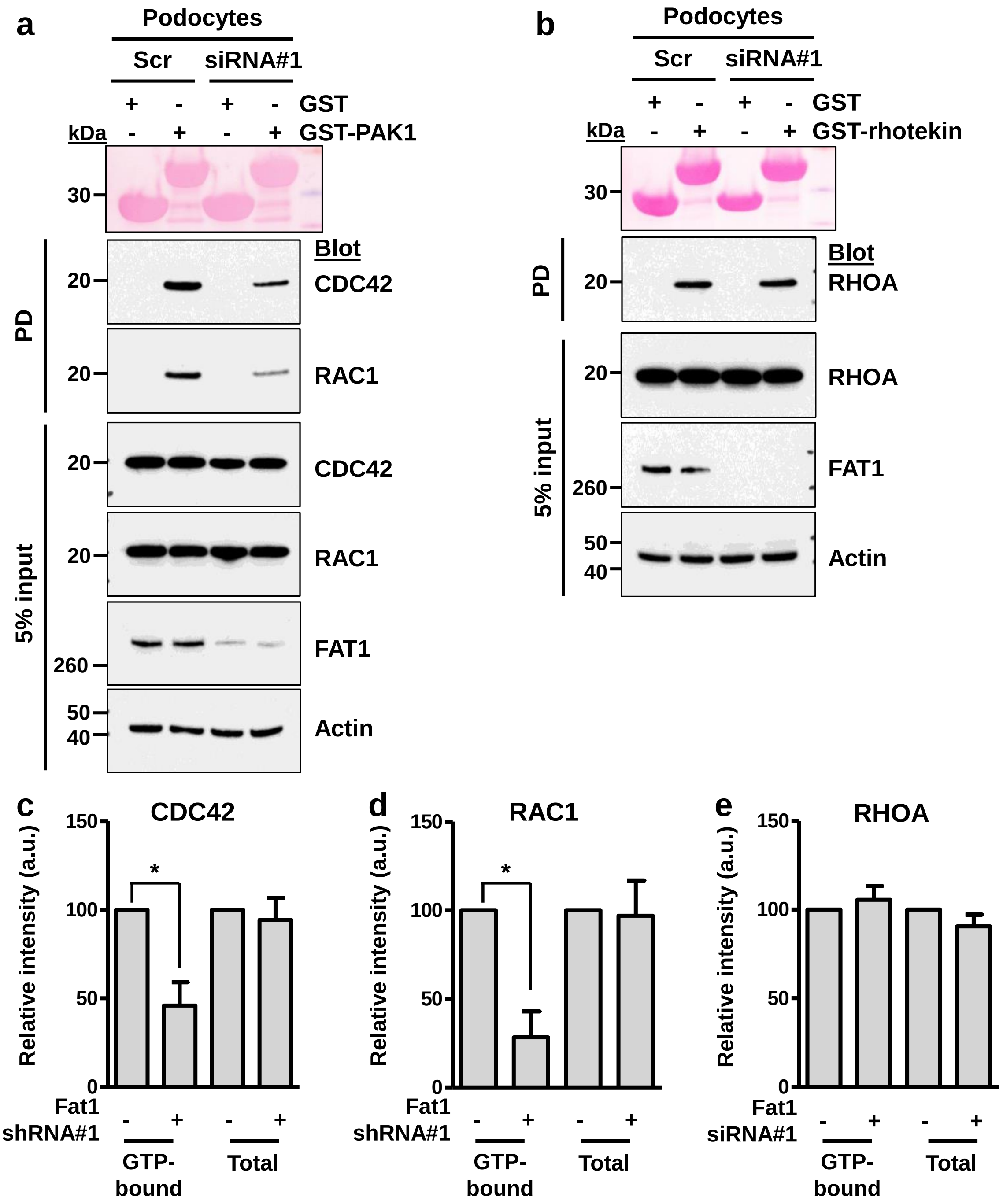

Supplementary Figure 3. Effects of FAT1 knockdown on RHO GTPases activity in differentiated cultured human podocytes.

(a) Active GTP-bound RAC1 and CDC42 precipitated from differentiated podocytes transfected with scrambled or FAT1 siRNA using a GST-PAK1 (CRIB) pulldown assays. Compared to control podocytes, podocytes transfected with FAT1 siRNA exhibited a significant decrease in relative RAC1 and CDC42 46% and 28%, respectively). The efficiency of knockdown by siRNA was confirmed by immunoblotting with an anti-FAT1 antibody (second to lowest panel). **a** and **b** represent 3 experiments each.

(b) Active GTP-bound RHOA precipitated from differentiated podocytes transfected with scrambled (Scr) or FAT1 siRNA using a GST-rhotekin (RBD) pulldown assay. Cells transfected with scrambled control siRNA versus FAT siRNA exhibited no significant difference in relative RHOA activity.

(c-e) Quantification of CDC42 (**c**), RAC1 (**d**), and RHOA (**e**) in FAT1-depleted cells compared to control cells. Error bars indicate the standard deviaion for three independent experments. * < 0.05, t-test.

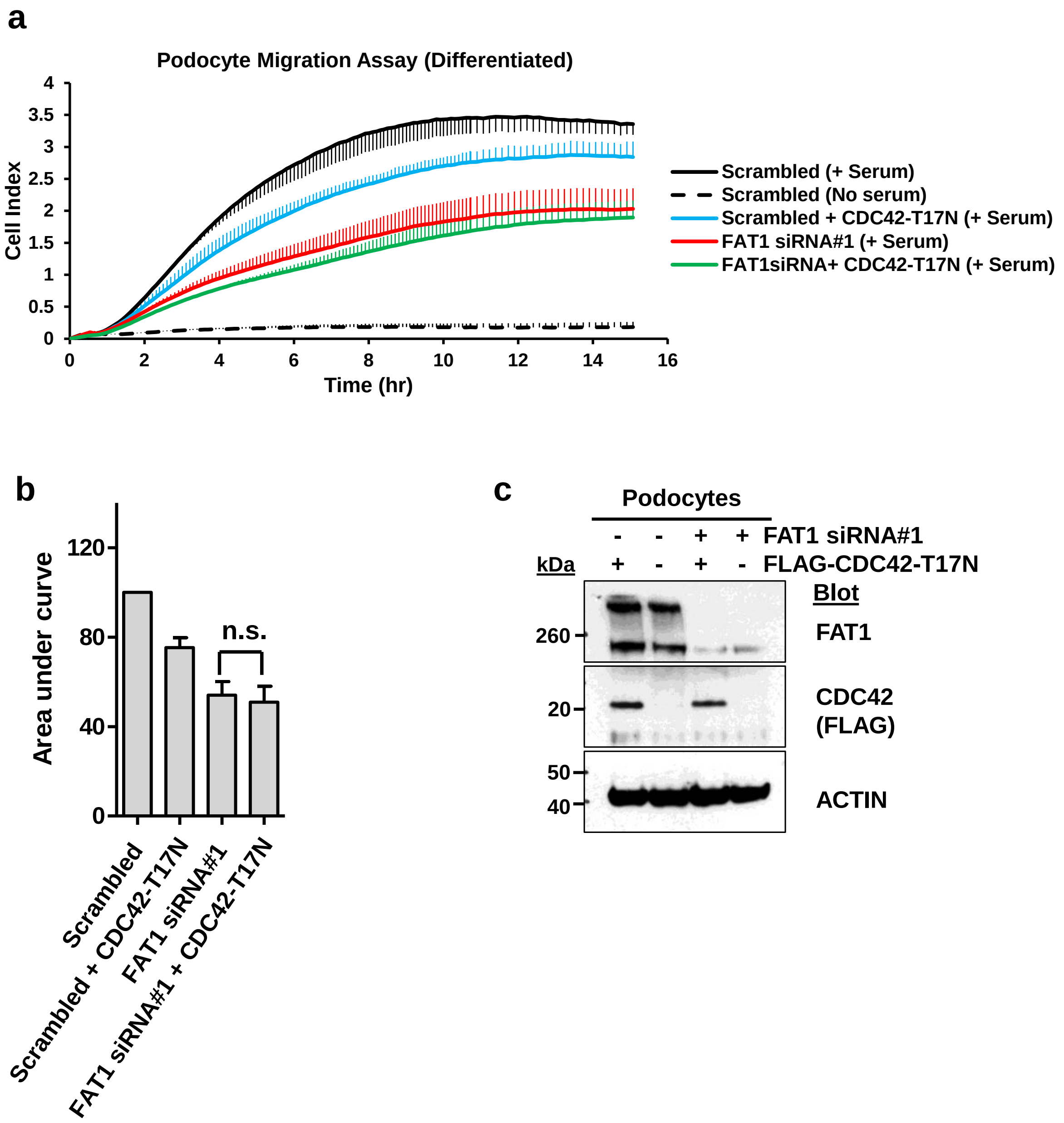

Supplementary Figure 4. Effects of dominant negative CDC42 and FAT1 knockdown on podocyte migration.

(a) To overexpress of CDC42-T17N in differentiated podocytes, pLenti-CMV-Blast-CDC42-T17N and lentiviral packaging vectors were used. Overexpression of CDC42-T17N resulted in decreased migration in differentiated podocytes (blue). However, overexpression of CDC42-T17N failed to further decrease migration in podocytes with FAT1 knockdown (red *vs.* green). Each cell index value corresponds to the average of more than triplicates and standard deviation is in only one direction for clarity.

(b) Bar graphs represent the area under curved of (**a**), and data represent the mean + stand deviation of three independent experiments. n.s., not significant, t-test.

(c) Western blot to examine efficiency of FAT1 knockdown and CDC42-T17N overexpression in differentiated cultured human podocytes. **c** represent 3 experiments.

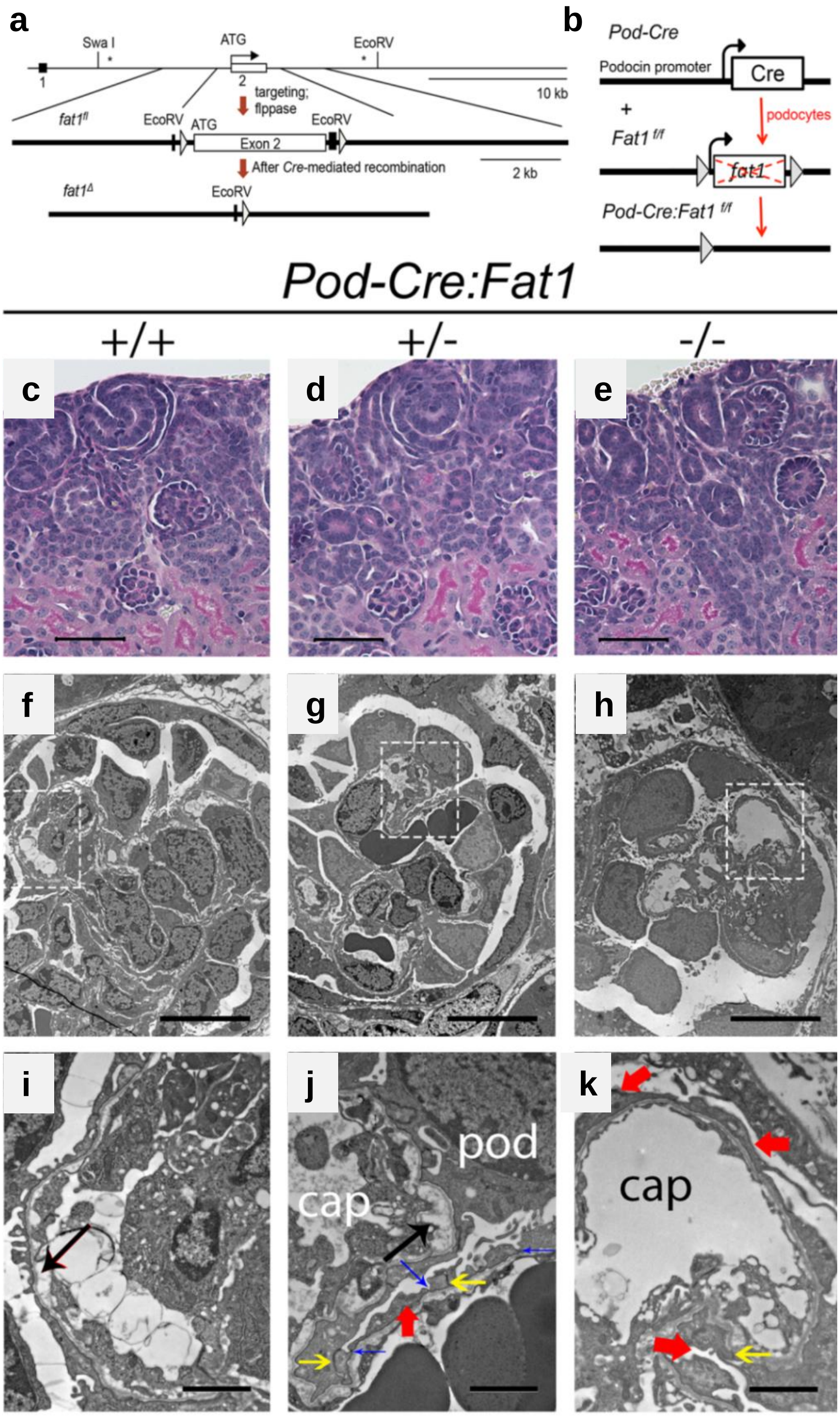

Supplementary Figure 5. Podocyte-specific *Fat1* deletion disrupts podocyte differentiation in developing mice.
(a) Conditional targeting strategy for the mouse *Fat1* locus. Asterisks indicate the locations of 5' and 3' external probes used for Southern analysis to confirm targeting. Neo selection cassette was removed by flippase-mediated recombination.
(b) Breeding *pod-Cre* and *Fat1f/f* mice results in podocyte specific *Fat1* deletion.
(c-e) PAS stains show normal nephron and glomerular development, and no significant difference in nephrogenic zone between wild type and *Fat1* mutants.
(f-h) Transmission electron micrograph of renal glomeruli from wild type and mutant mice.
(i-k) Inset of panels **f-h**.
(f, i) Wild type glomeruli show normal ultrastructure including split immature GBM (arrow).
(j) $Fat1^{+/-}$ glomeruli show foot process effancement (red arrow), tight junctions (yellow arrows) and some slit diaphragms (blue arrows).
(k) $Fat1^{-/-}$ glomeruli show complete foot process effancement (red arrows), normal endothelium and GBM. Scale bars = 10 μm (d-e) and 2 μm (g-i). Pod, podocyte; cap, capillary.

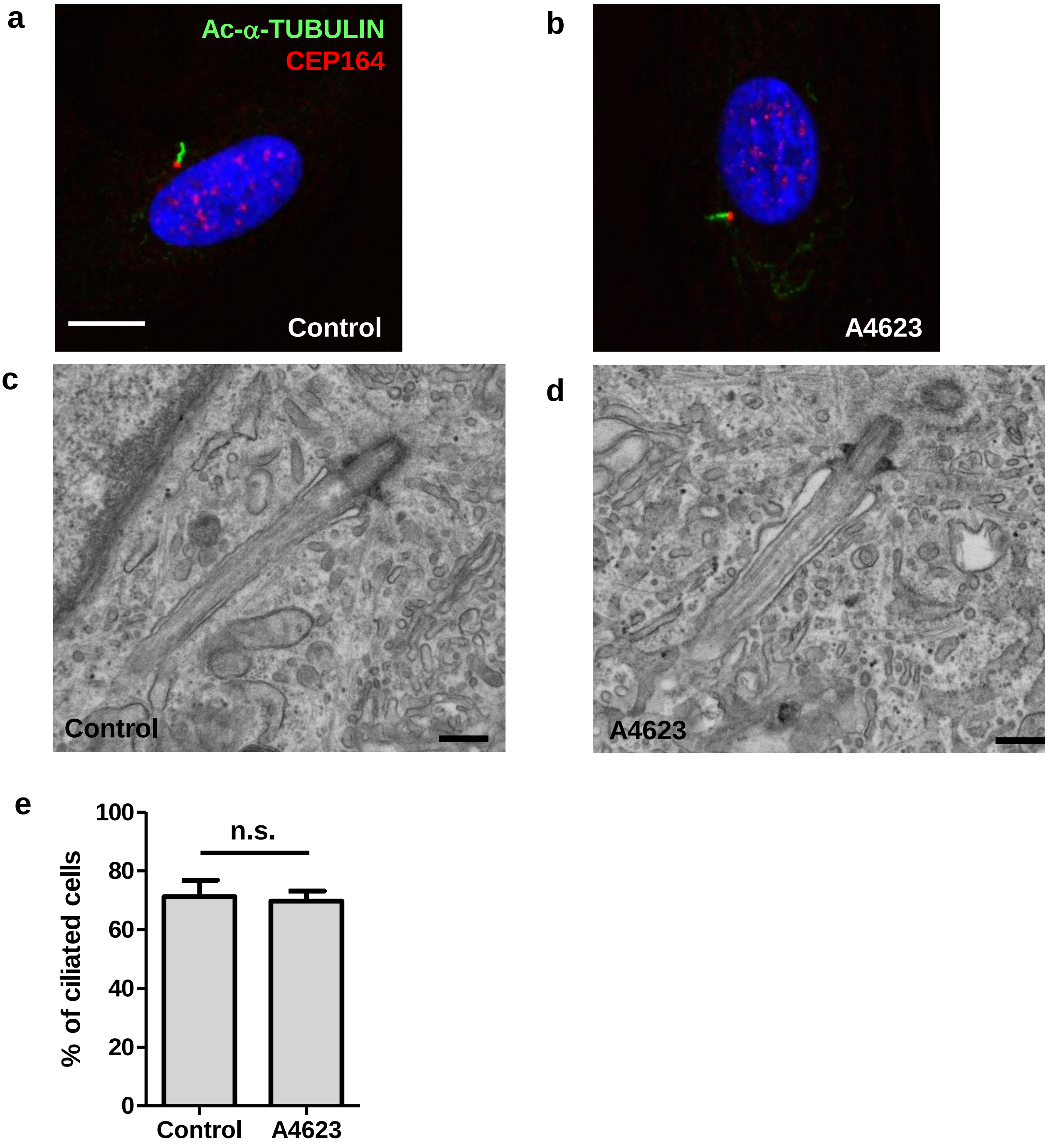

Supplementary Figure 6. A truncating mutation in *FAT1* does not cause defects in cilia.
(a,b) Fibroblasts from control and affected individuals A4623 were grown on coverslips, serum starved for 48 hr, then fixed and processed for immunofluorescence with antibodies against acetylated-α-tubulin and CEP164 antibodies to stain ciliary axoneme and centrosome, respectively. There was no defect in ciliation. Scale bar: 10 μm.
(c,d) Transmission electron microscopy (TEM) images of fibroblasts from healthy control and A4623 who carries a homozygous c.3093_3096del:p.P1032Cfs*11 mutation in *FAT1*. Fibroblasts were serum-starved for 48 hours before proceeding for TEM. Note that the cilium in A4623 fibroblasts does not have any appreciable structural defect. Scale bar: 0.5 μm.
(e) The ability of cells to form cilia, in cells with a unique rod like acetylated-a-tubulin staining was quantified from images. Graphs show the mean + standard deviation of three independent experiments, in which at least 60 cells were counted. n.s., not significant, t-test.

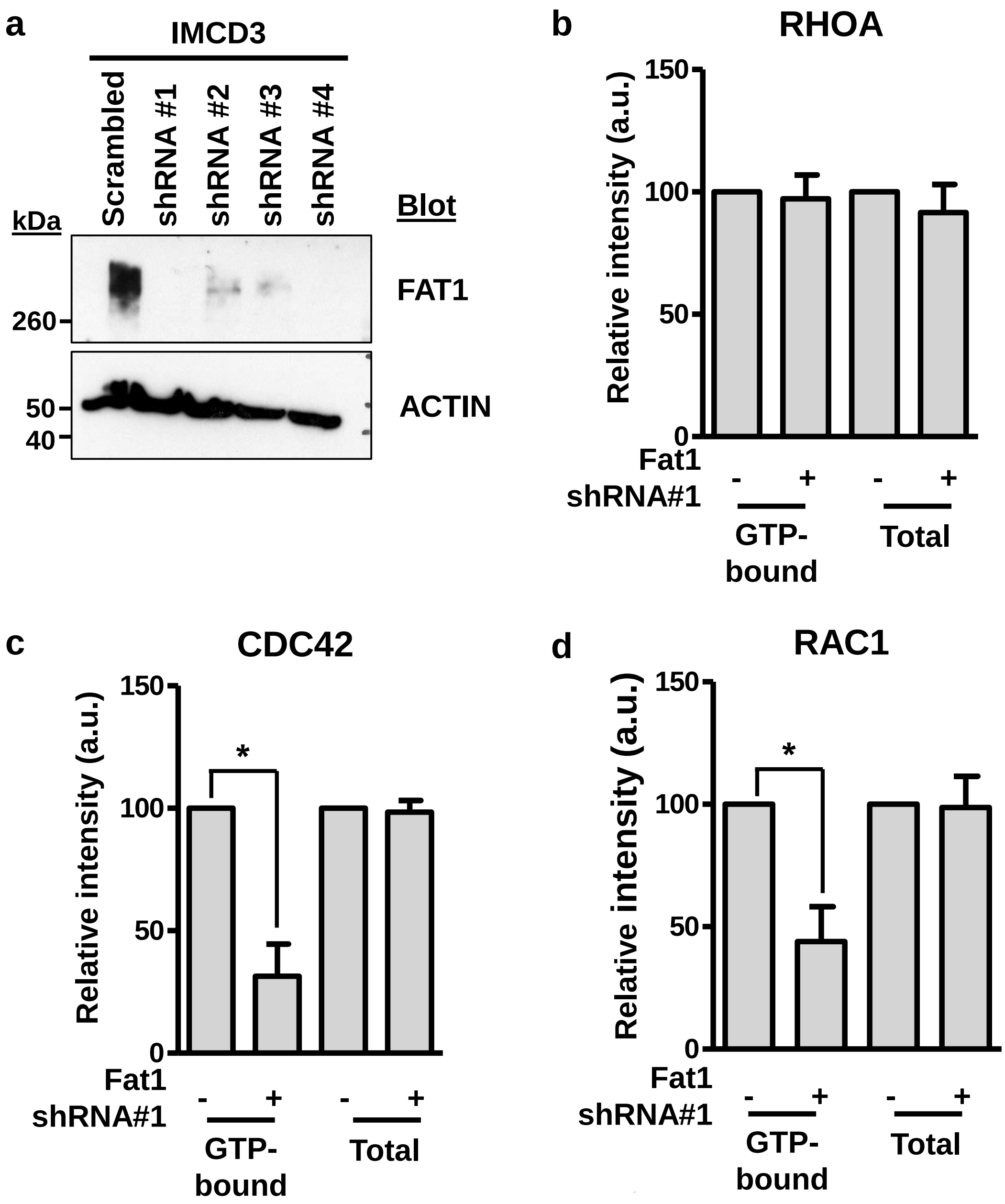

Supplementary Figure 7. Effects of *Fat1* knockdown on RHO GTPase activity in IMCD3 cells.

(a) Stable knockdown of *Fat1* in IMCD3 using lentivirus-mediated shRNAs demonstrates efficient knockdown by all four shRNAs.

(b-c) Quantification of RHOA (**b**), CDC42 (**c**), and RAC1 (**d**) in *Fat1*-depleted IMCD3 cells compared to control cells. Error bars indicate the standard deviaion for three independent experments for RHOA and four independent experiment for CDC42 and RAC1. * < 0.05, t-test.

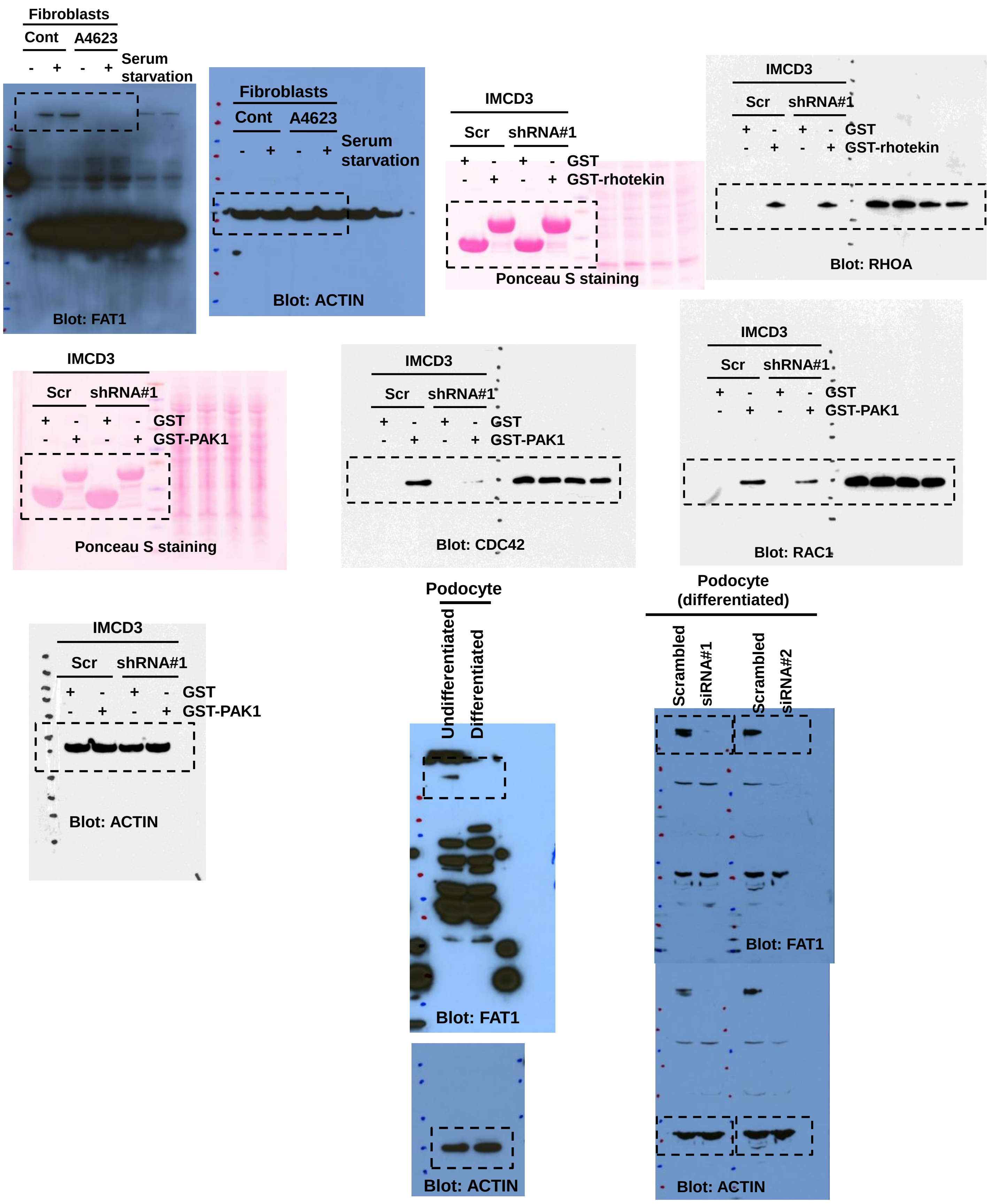

Supplementary Figure 8a. Full scans of Western blot data corresponding to Fig 2a, 4e, 4f, Supplementary Fig. 2a and 2c. .

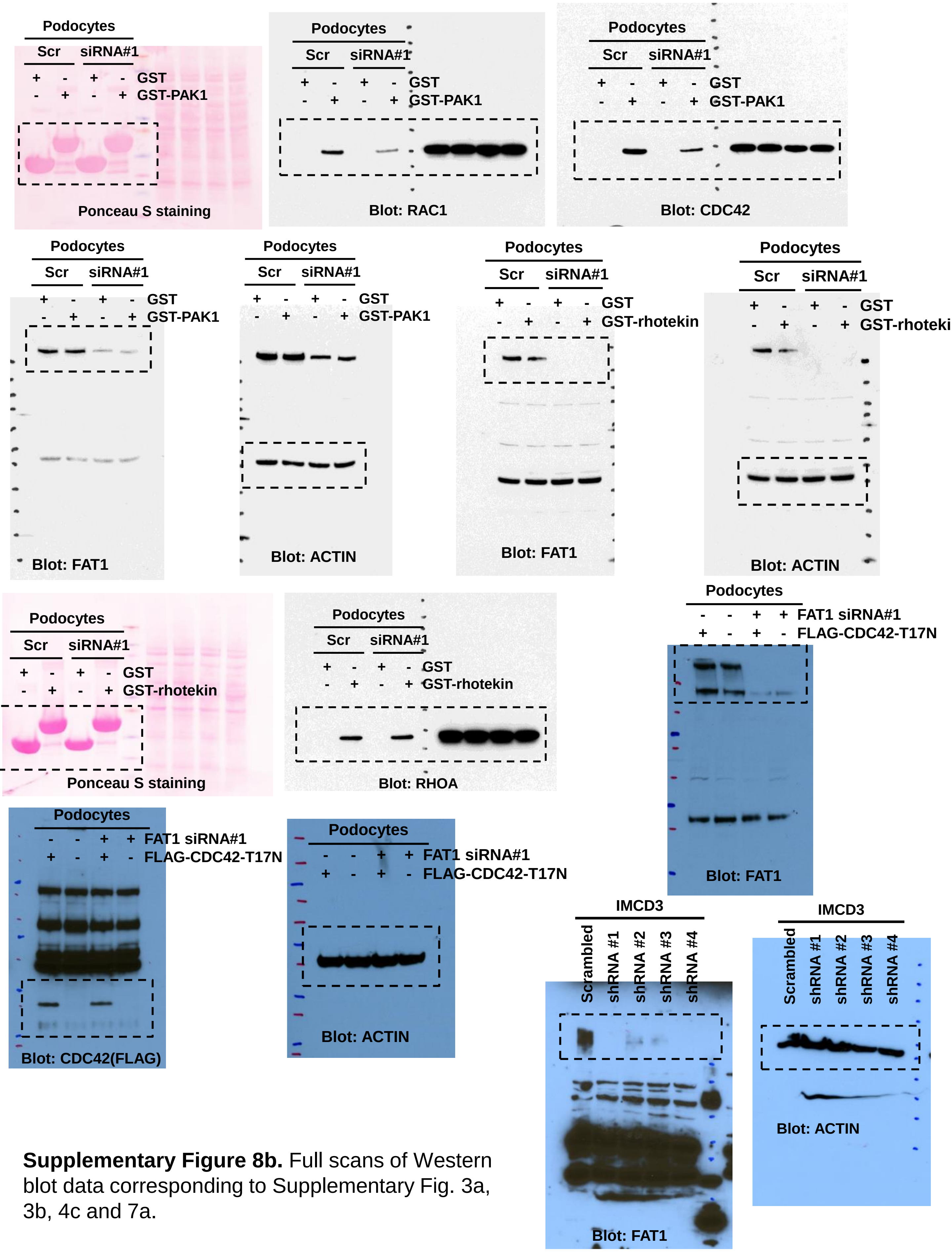

Supplementary Figure 8b. Full scans of Western blot data corresponding to Supplementary Fig. 3a, 3b, 4c and 7a.

Supplementary Table 1. Filtering process for variants from normal reference sequence (VRS) following whole exome sequencing (WES) in A4623 and A3027

FAMILY	A4623	A3027
Consanguinity	Yes	Yes
[a]# of homozygosity peaks	15	18
Cumulative Homozygosity by descent[a] [Mb]	151.1	215.5
[b]Hypothesis from mapping: homozygous or heterozygous	homozygous	homozygous
Total sequence reads (Million)	144	49
Matched Reads	98.4%	99.2%
Total DIPs	52,571	10,779
Exonic DIPs	244	262
% exonic / total DIPs	0.46%	2.40%
DIPs in homozygosity peaks	17	22
DIPs which are not SNP137	17	20
DIPs after inspection and which are not SNP137 (>1% MAF)	2	2
Sanger confirmation / Segregation	2	0
Total SNPs	430,162	30,745
Exonic SNPs	5,194	1,364
% exonic / total SNPs	1.21%	4.40%
SNPs in homozygosity peaks	109	186
SNPs which are not SNP137	68	136
SNPs after inspection and which are not SNP137 (>1% MAF)	12	8
Sanger confirmation / Segregation	1	2
Causative gene	***FAT1***	***FAT1***
Mutation effect on gene product	c.3093_3096del; p.Pro1032Cysfs*11 (homozygous)	c.857A>G; p.Asn286Ser (homozygous)

[a]see Fig. 1 for A4623.

[b]evaluation for homozygous variants was done in regions of homozygosity by descent for A4623.

"-" , not applicable; DIP, deletion/insertion polymorphism; SNPs, single nucleotide polymorphism

Supplementary Table 2. Oligonucleotide sequences used in this study

**Target sequences of siRNAs**#	
FAT1 siRNA	GGAAGGAATTGGAATCGTT
FAT1 siRNA#2	CAAGATGGGTGTTTACATT
FAT1 siRNA	TCACATGATTGGAGTAATA
FAT1 siRNA	CAAGGAAGCTCGTTCACAA
**Target sequences of shRNAs**	
Fat1 shRNA#1	GCCGAGGGAATAGGTATTATT
Fat1 shRNA #2	CCACCCAATTACTCAGTGAAA
Fat1 shRNA #3	GCCACGTCATTCGCTATGTTA
Fat1 shRNA #4	CGCCACGATTACTCTCAATAA

#For FAT1 siRNA#1, a pool of the listed four siRNAs was transfected into cultured podocytes, whereas for FAT1 siRNA#2, a single siRNA was transfected.
